# Measuring Life Satisfaction in Parkinson’s Disease and Healthy Controls Using the Satisfaction With Life Scale

**DOI:** 10.1371/journal.pone.0163931

**Published:** 2016-10-24

**Authors:** Lise Løvereide, Peter Hagell

**Affiliations:** 1 The Norwegian Centre for Movement Disorders, Stavanger University Hospital, Stavanger, Norway; 2 The PRO-CARE Group, School of Health and Society, Kristianstad University, Kristianstad, Sweden; University of the West Indies Faculty of Medical Sciences Mona, JAMAICA

## Abstract

The 5-item Satisfaction With Life Scale (SWLS) was designed to measure general life satisfaction (LS). Here we examined the psychometric properties of the SWLS in a cohort of persons with Parkinson`s disease (PwPD) and age and gender matched individuals without PD. The SWLS was administered to PwPD and controls from the Norwegian ParkWest study at 5 and 7 years after the time of diagnosis. Data were analysed according to classical test theory (CTT) and Rasch measurement theory. CTT scaling assumptions for computation of a SWLS total score were met (corrected item-total correlations >0.58). The SWLS was reasonably well targeted to the sample and had good reliability (ordinal alpha, 0.92). The scale exhibited good fit to the Rasch model and successfully separated between 5 statistically distinct strata of people (levels of SWLS). The seven response categories did not work as intended and the scale may benefit from reduction to five response categories. There was no clinically significant differential item functioning. Separate analyses in PwPD and controls yielded very similar results to those from the pooled analysis. This study supports the SWLS as a valid instrument for measuring LS in PD and controls. However, Rasch analyses provided new insights into the performance and validity of the SWLS and identified areas for future revisions in order to further improve the scale.

## Introduction

Parkinson’s disease (PD) is associated with a number of motor and non-motor symptoms that have a major influence on the lives of those affected by the disease and how satisfied they are with their lives [1]. Greater understanding of life satisfaction (LS) is necessary for improving PD management, particularly from a person-centred chronic disease management perspective, which in turn requires valid tools to quantify LS.

One of the most frequently used LS scales is the generic 5-item Satisfaction With Life Scale (SWLS). The SWLS was developed to measure people’s perception and evaluation of their overall LS [2] Although the SWLS has been extensively tested in different populations [3–8] there are still some concerns regarding its psychometric properties. For example, whereas the three first SWLS items represent the present, the two last items represent the past and the last item in particular has been suggested to be relatively weakly associated with the other items [7]., thus challenging the unidimensionality and internal integrity of the scale. Furthermore, while its generic nature should allow for comparison of scores between respondent groups such as various patient populations and control subjects, the extent to which this is supported empirically appears untested.

Recent studies have suggested that the SWLS is useful for measuring LS in persons with PD (PwPD) [5–8]. Whereas these studies used classical test theory (CTT) methodology based on parametric statistics, this approach does not take the ordinal nature of data into account. Furthermore, modern psychometric methods and in particular the Rasch measurement model, is considered superior to CTT and provides more detailed insights into the psychometric properties of rating scales, including score invariance between subgroups of people [9].

The aim of this study was to examine the psychometric properties of SWLS in a cohort of PwPD and age matched individuals without PD using CTT and the Rasch measurement model.

## Materials and Methods

### Patients and controls

This paper is based on the Norwegian ParkWest study [10]. PwPD were included when diagnosed with PD according to the UK-Brain bank criteria [11], and the control group was recruited among their relatives and friends and from social clubs for elderly. Exclusion criteria for the control group were parkinsonism at clinical examination and/or inability to complete the study program at baseline. The ParkWest cohort has been followed prospectively bi-annually from the time of diagnosis. The SWLS has been used at the 5 (time 1; T1) and 7 (time 2; T2) year visits after diagnosis. Out of 165 PwPD and 170 controls at the 5-year visit and 147 PwPD and 155 controls at the 7-year visit, this study included 146 PwPD and 163 controls included from T1 and 116 PwPD and 143 controls from T2 that had responded to the SWLS. Table 1 shows clinical and demographic data from the 5-year visit (T1). The study was approved by the Regional Committee for Medical and Health Research Ethics in Western Norway. All participants provided written informed consents.

**Table 1:** Clinical and demographic features of people with PD (PwPD) and controls at T1 (5 years following the diagnosis of PD).

	PwPD (n = 146)	Controls (n = 163)
Male / Female, n (%)	89 (61) / 57 (39)	88 (54) / 75 (46)
Age (years), mean (SD)	66 (9)	65 (9)
Education (years), mean (SD)	11 (3)	12 (3)
Hoehn & Yahr stage, median (q1-q3)	2 (1–5)	-
UPDRS III (motor examination), median (q1-q3)	22 (4–62)	-
MMSE, median (q1-q3)	27(10–30)	29 (22–30)

PD, Parkinson’s disease; SD, standard deviation; UPDRS, Unified Parkinson`s Disease Scale; MMSE, Mini-Mental State Examination.

### Examinations

The full protocol and study procedures have been described in detail elsewhere [10]. Demographic data included in this study were sex, age and years of education. Disease severity was assessed using the Hoehn &Yahr staging [12] and part III (motor examination) of the Unified PD Rating Scale (UPDRS) [13]. Due to ethical considerations, the data will not be shared publicly when data may compromise the privacy of study participants. An ethically compliant data set will be made available to interested researchers on request to the authors.

### Satisfaction with Life Scale (SWLS)

The SWLS consists of 5 items with seven response categories each that are scored from 1–7, where 1 = strongly disagree and 7 = strongly agree. Summation of complete item responses yield a total raw score that can range from 5 to 35 [2]. A total score of 20–24 is considered average LS; total scores between 25–29 are considered high LS and scores between 15–19 are slightly below average, whereas scores of 5–14 and 30–35 suggest extremely low or high LS, respectively. This study used the Norwegian version of the SWLS (http://internal.psychology.illinois.edu/~ediener/SWLS.html).

### Analyses

The SWLS was analysed psychometrically according to both CTT and the Rasch measurement model using IBM SPSS version 22 (IBM Corp., Armonk, NY), R version 3.2.2 (“psych” package version 1.5.8; www.r-project.org), FACTOR version 10.3.01 (http://psico.fcep.urv.es/utilitats/factor/), Stata MP version 14.1 (StataCorp, College Station, TX, USA), and RUMM2030 (Professional Edition, version 5.4) [14]. CTT analyses were designed to replicate the previous evaluation of the SWLS in PD [8]. In contrast to previous CTT analyses of the SWLS in PD, we also included data from healthy controls and the ordinal nature of raw item data was taken into account in the analyses.

SWLS data were analysed regarding completeness (percentages of complete item responses and computable total scores), scoring assumptions for the legitimacy of computing summed total scores (i.e., similar item score means and standard deviations (SD); corrected item-total correlations ≥0.30 and ≥0.40 suggesting sufficient contribution by each item to the total score and unidimensionality, respectively). Unidimensionality was further tested by exploratory factor analyses (EFA) using minimum rank factor analysis based on polychoric correlations and parallel analysis (500 random permutations of raw data) to determine the number of dimensions [15]. Further analyses included targeting (i.e., average total SWLS scores close to the scale midpoint of 20; floor/ceiling effects ≤15%; skewness ≤ ±1), and reliability (i.e., coefficient alpha ≥0.80), including the standard error of measurement (SEM = SD x √1-reliability) and the smallest detectable difference (SDD = SEM x 1.96 x √2). All analyses were conducted with the pooled (PwPD + controls) sample, as well as for PwPD and controls separately. To account for the ordinal nature of item level data, item-total correlations were computed based on polychoric correlations, reliability was assessed by the ordinal version of coefficient alpha [16], and SEM and SDD were calculated based on ordinal alpha. Traditional parametric item-total correlations and coefficient alpha were also computed for comparative reasons. For methodological details regarding these analyses, see [8, 15–17].

Analyses were also extended to include the Rasch measurement model, which mathematically defines what is required from rating scale item level data to conform with linear measurement [18]. According to this model, the probability of a certain item response is a function of the difference between the level of the measured construct (e.g., LS) represented by the item and that reported by the person. The model separately locates persons and items on a common linear logit (log-odd units) metric, ranging from minus to plus infinity (with mean item location set at zero). If data accord sufficiently with the model, linear measurement and invariant comparisons are possible [17, 19–22].

Here we used Rasch analysis to address targeting, reliability, Rasch model fit, rating scale response category functioning, and uniform and non-uniform Differential Item Functioning (DIF) by time of assessment (T1 vs. T2), group (PwPD vs. controls), gender, age, and (for PwPD only) UPDRS III motor score. Subgroups for DIF analyses of age and UPDRS III were defined according to their respective median values. DIF by time of assessment was checked at the outset of these (and the CTT) analyses and absence of DIF by time was taken as support for merging data from the two time points, thereby gaining precision of estimates [23]. Following the main analysis, data were also Rasch analysed separately for PwPD and controls.

Rasch analyses were conducted using the unrestricted polytomous (“partial credit”) model as implemented in RUMM2030 (Professional Edition version 5.4) [14], with the sample divided into eight class intervals (subgroups with similar levels of LS according to SWLS total scores). Analyses include both graphical and statistical methods, which are of equal primacy. To facilitate comparisons between analyses (full sample vs. PwPD vs. controls) and because type I errors increase by increasing sample sizes, data were analysed with the effective sample size algebraically adjusted to n = 250 in the calculation of P-values, while leaving all other aspects of data (e.g., locations, fit residuals) unaltered [20, 24, 25]. Bonferroni adjustments for multiple null hypothesis testing were applied (alpha level of significance, 0.05) [25, 26].

## Results

**Fig 1:**
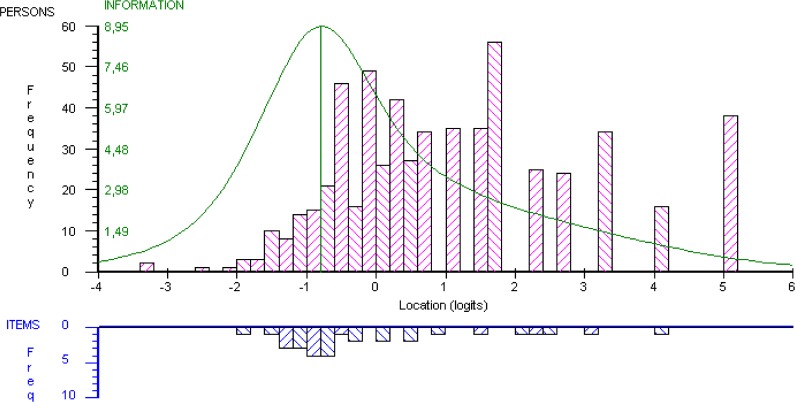
Distribution of locations of people and SWLS response category thresholds. Distribution of locations of people (pooled sample of people with PD and control subjects; upper panel) and SWLS response category thresholds (lower panel) on the common logit metric (x-axis; positive values = higher life satisfaction). All locations are relative to the mean item threshold location, which is set at 0 logits. Thresholds are the scale’s points of measurement and represent locations where there is a 50/50 probability of responding in either of two adjacent item response categories. There is thus one threshold less than the number of response categories for each item, rendering 5 items x (7 response categories-1) = 30 thresholds (points of measurement) for the SWLS. Superimposed on the person distribution graph is the information function curve (the inverse of measurement error; higher values = less error and more information in scores, i.e., better measurement precision). Maximum information (vertical line under the information function curve) corresponds to a location of -0.8 logits (representing a raw SWLS score of 16–17 on the original 5–35 score range). PD, Parkinson’s disease; SWLS, Satisfaction with Life Scale.

We found no evidence of DIF by time (P>0.34). Therefore, the main analyses were conducted with the merged (T1+T2) data set. Table 2 reports results from the CTT analyses. We found support for all aspects assessed, including CTT scoring assumptions, targeting and reliability in the pooled as well as the separate analyses of PwPD and control subjects. The SWLS was reasonably well targeted to the sample, although the scale tended to represent lower levels of LS compared to those reported by the sample, as suggested by average raw total scores above the scale midpoint and a slight negative skew (Table 2). Fig 1 provides a more detailed account of targeting as derived from Rasch analysis. It is seen that the scale represents a quantitative continuum from lower to higher levels of LS (ranging approximately 5.8 logits, from about -1.83 to 4.01 logits; Fig 1, lower panel) that is similar to that found in the sample (ranging approximately 8.4 logits, from about -3.23 to 5.14 logits; Fig 1, upper panel). The mean person location is 1.07 logits, i.e., the sample reported LS levels on average about 1 logit above that represented by the SWLS. It is also seen that there tends to be gaps in the scale’s representation of the variable at levels around 1 logit and above (Fig 1, lower panel). As a consequence, people with higher levels of LS are measured with less precision, as illustrated by relatively low information function values (i.e., the inverse of measurement error) at levels above about 1 logit (as well as below about -2 logits; Fig 1, upper panel). However, reliability was good and the scale was able to separate between 5 statistically distinct strata of people (Table 3).

**Table 2:** Descriptive and psychometric statistics according to classical test theory (CTT) for raw SWLS scores (possible score range, 5–35) from PwPD and control subjects.

	PwPD & controls	PwPD	Controls
*Data completeness*			
Range of missing item responses, %	7.6–7.7	12.2–12.6	3.2–3.2
Computable scale scores, %	91.9	86.7	96.8
*Scaling assumptions*			
Item mean scores, min-max ^a^	4.7–5.6	4.1–5.2	5.2–5.9
Item SD, min-max	1.3–1.6	1.4–1.7	1.1–1.4
Corrected polychoric item-total correlation, min-max ^b^	0.67–0.91	0.66–0.89	0.68–0.92
Corrected Pearson item-total correlation, min-max ^b^	0.59–0.83	0.57–0.81	0.58–0.82
*Item EFA (MRFA)*			
F1 loadings (min-max)	0.66–0.91	0.63–0.87	0.66–0.89
F1 / F2% common variance explained	77.6 / 13.0	75.5 / 15.0	79.9 / 10.1
F1 / F2% common variance explained from PA	54.2 / 37.2	53.5 / 36.3	52.6 / 35.6
*Targeting*			
Total score, mean (SD) ^c^	25.7 (6.5)	23.4 (6.7)	27.7 (5.5)
Total score, median (q1-q3) ^c^	27 (22–30)	24 (19–28)	29 (25–32)
Total score, min-max ^d^	5–35	5–35	5–35
Total score floor/ceiling effects, % ^e^	0.3/6.6	0.4/3.1	0.3/9.6
Total score skewness ^f^	-0.7	-0.4	-1.0
*Reliability*			
Ordinal alpha ^g^	0.92	0.89	0.92
Coefficient alpha ^g^	0.89	0.87	0.89
SEM, ordinal alpha based (% of range) ^h^	1.8 (6.1)	1.9 (6.4)	1.8 (6.1)
SEM, alpha based (% of range) ^h^	2.2 (7.1)	2.4 (8.1)	1.8 (6.1)
SDD, ordinal alpha based (% of range) ^i^	5.1 (16.9)	5.3 (17.6)	5.1 (16.9)
SDD, alpha based (% of range) ^i^	5.9 (19.6)	6.7 (22.5)	5.1 (16.9)

^a^ Item scores range from 1 to 7, higher scores represent more satisfaction.

^b^ Should be ≥0.30–0.40 [17].

^c^ Should be close to scale midpoint (i.e., 20) [17].

^d^ Should span most of scale range (i.e., 5–35) [17]

^e^ Should be ≤15% [17].

^f^ Should be ≤ ±1 [17].

^g^ Should be ≥0.80 [17].

^h^ Computed based on ordinal / regular coefficient alpha (SD x √1-alpha) [17].

^i^ Computed from the SEM based on ordinal / regular coefficient alpha (SEM x 1.96 x √2) [8].

SWLS, Satisfaction With Life Scale; PD, Parkinson’s disease; SD, standard deviation; EFA, exploratory factor analysis; MRFA, minimum rank factor analysis; F, factor; PA, parallel analysis; SEM, standard error of measurement; SDD, smallest detectable difference.

**Table 3:** Overall Rasch model fit statistics, reliability and targeting of the SWLS among PwPD and controls^a^.

	PwPD & Controls	PwPD	Controls
*Targeting*			
Person location, mean (SD) ^b^	1.07 (1.74)	0.48 (1.41)	1.71 (1.97)
*Reliability*			
Person separation index ^c^	0.86	0.85	0.85
Strata ^d^	5.64	4.53	6.20
*Overall Rasch model fit*			
Item fit residual, mean ^e^ (SD ^f^)	-0.59 (3.58)	0.02 (1.87)	-0.65 (2.83)
Item residual correlations ^g^	≤0.106	≤0.122	≤0.072
Person fit residual, mean ^e^ (SD ^f^)	-0.69 (1.26)	-0.59 (1.23)	-0.68 (1.20)
Total item-trait interaction, chi-square (df)	48.78 (35)	40.38 (20)	42.82 (20)
P-value ^h^^.^^i^	0.365	0.027	0.013

^a^ As analysed with the sample divided into eight class intervals according to person locations on the measured variable.

^b^ Relative to the mean item logit location (i.e., zero).

^c^ Analogous to Cronbach’s alpha [27].

^d^ Number of statistically distinct groups (separated by ≥3 standard errors) that can be distinguished by the scale [22].

^e^ Should be close to 0 [20].

^f^ Should be close to 1 [20].

^g^ Should be <0.30 [17].

^h^ Should be >0.05 [20].

^i^ Bonferroni corrected for multiple testing.

SWLS, Satisfaction With Life Scale; PwPD, People with Parkinson’s disease; SD, standard deviation; df, degrees of freedom.

**Fig 2:**
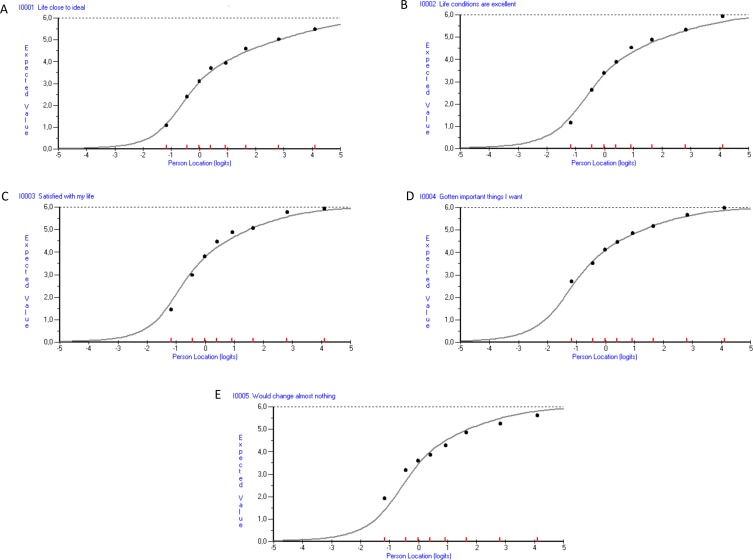
Item characteristic curves (ICCs) of the five SWLS items. ICCs representing SWLS items 1 (panel A), 2 (panel B), 3 (panel C), 4 (panel D) and 5 (panel E). Grey curves (ICCs) represent expected item responses (y-axis) for each person location (x-axis) on the life satisfaction continuum (positive values = higher life satisfaction). Black dots represent the observed responses from groups of people at similar locations on the measured continuum (x-axis). ICC, Item characteristic curve; SWLS, Satisfaction with Life Scale.

Item response data displayed acceptable overall fit to the Rasch model (Table 3). Item characteristic curves (ICCs) of empirical responses among people in the eight class intervals relative to Rasch model expectations showed negligible to modest discrepancies (Fig 2). Item 5 had the poorest accordance between empirical data and model expectations, where empirical responses tended to exhibit a less steep pattern than expected, suggesting that this item may represent a somewhat different construct than the scale as a whole. Statistically, this was mirrored by a relatively large positive fit residual and a significant chi-square value (Table 4).

**Table 4:** Rasch item and fit statistics^a^.

Items ^b^			Item statistics ^c^		Fit statistics		
No.	Contents		Location	SE	Residual ^d^		Chi square ^e^
*PwPD & Controls*							
4	So far I have gotten the important things I want in life		-0.529	0.048	-0.275		3.096
3	I am satisfied with my life		-0.193	0.044	**-4.575**		15.037
5	If I could live my life over, I would change almost nothing		0.017	0.042	**4.880**		18.573
2	The conditions of my life are excellent		0.163	0.043	**-2.870**		8.552
1	In most ways my life is close to my ideal		0.541	0.044	-0.103		3.528
*PwPD*							
4	So far I have gotten the important things I want in life		-0.535	0.063	0.579		1.638
5	If I could live my life over, I would change almost nothing		-0.183	0.055	2.488		11.595
3	I am satisfied with my life		-0.130	0.059	-2.260		**14.447**
2	The conditions of my life are excellent		0.253	0.058	-1.365		6.724
1	In most ways my life is close to my ideal		0.594	0.058	0.664		5.980
*Controls*							
4		So far I have gotten the important things I want in life	-0.493	0.075	-0.528	3.068	
3		I am satisfied with my life	-0.245	0.069	**-3.931**	10.424	
2		The conditions of my life are excellent	0.026	0.069	-2.006	4.226	
5		If I could live my life over, I would change almost nothing	0.274	0.064	**3.747**	**13.994**	
1		In most ways my life is close to my ideal	0.438	0.070	-0.553	11.106	

^a^ Performed with the sample divided into eight class intervals according to person locations on the measured variable.

^b^ Listed in order of location across the latent continuum from lower (negative values) to higher (positive values) life satisfaction.

^c^ Item locations are the mean of each item’s response category threshold values expressed in linear log-odds units (logits), with mean item location for the whole scale set at 0.

^d^ Standardized residuals summarise the deviation of observed from expected responses. Deviation from the recommended [20] range of -2.5 to +2.5 are bold.

^e^ Bonferroni corrected statistically significant deviations across class intervals, suggesting item misfit, are bold.

SE, standard error; PwPD, People with Parkinson’s disease.

Given that items 4 and 5 have been suggested to represent a somewhat different construct than items 1–3, we conducted a principal component analysis (PCA) on the residuals in order to explore this issue. In agreement with previous hypothesis, these item groups loaded in different directions. Items 1–3 displayed negative loadings (-0.493 to -0.680) on the first principal component, whereas items 4 and 5 loaded positively (0.539 and 0.810, respectively). However, the two subsets did not yield significantly different person location estimates for more than 5.6% of individuals (binomial 95% Agresti-Coull CI, 3.3–9.2%), suggesting sufficient unidimensionality across all five items (as also suggested by the polychoric based EFAs; Table 2). Furthermore, item residual correlations of the full 5-item SWLS were low (Table 3), suggesting local independence.

**Fig 3:**
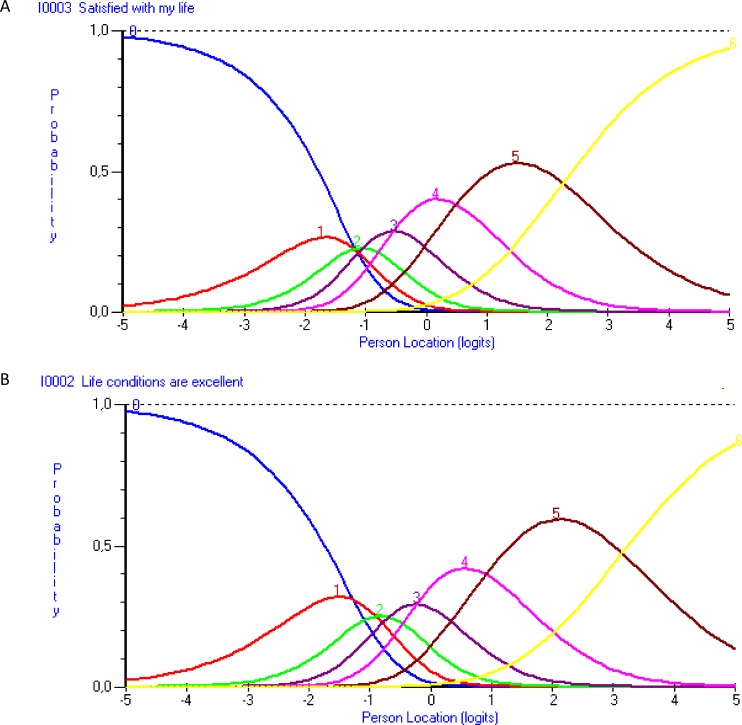
Example category probability curves. Locations on the life satisfaction continuum are indicated on the x-axis (with threshold locations centred at zero; positive values = higher life satisfaction) and the y-axis represents the probability of affirming response categories 1 through 7 (rescored as 0 through 6 in the analysis) relative to the location on the measured construct (x-axis). Panel A shows item 3, representing the pattern with disordering between the two first response category thresholds (also seen with item 1). Panel B displays item 2, representing items without disordered response category thresholds (also seen with items 4 and 5). Thresholds are the locations where there is a 50/50 probability of responding in either of two adjacent item response categories. SWLS, Satisfaction with Life Scale.

Assessment of the empirical functioning of the seven response categories showed that these did not work as expected with items 1 and 3. Specifically, the second and third response categories were problematic and while they did behave as expected with items 2, 4 and 5, the pattern was similar also among these items (Fig 3). That is, it appears difficult for people to distinguish between 7 levels of LS, particularly at the lower end of the continuum. We therefore explored reducing the number of response categories by post-hoc collapsing of the seven original response categories (scored as 0123456 in the analysis) into a five-category response scale (scored as 0011234) across all five items. Reanalysis did not reveal any problems with the revised response format while reliability was unaffected (0.86), suggesting that a five-category response scale may be advantageous. However, this needs empirical prospective confirmation and the overall fit deteriorated somewhat (overall item-trait chi-square interaction, 63.57; P = 0.013) following collapsing of response categories across items. This may be due to the collapsing of response categories that actually did work [28].

**Fig 4:**
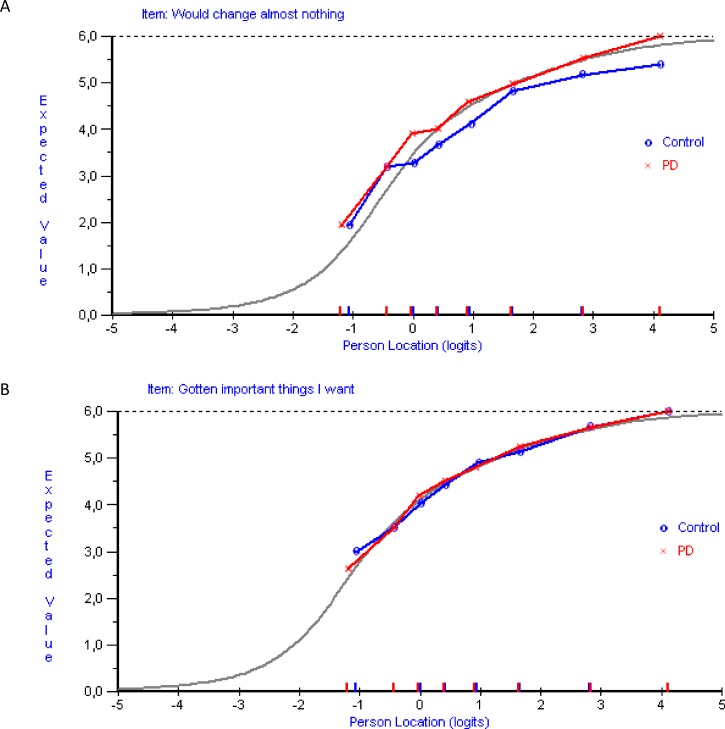
Differential Item Functioning (DIF) between people with PD and control subjects. Panel A displays uniform Differential Item Functioning (DIF) between people with PD and control subjects for item 5 of the SWLS. The item characteristic curve (ICC; grey curve) represents the expected response category endorsement (y-axis) at various levels of life satisfaction (x-axis). Superimposed plots represent the observed responses by people with PD (x) and control subjects (⭕), as ivided into eight class intervals according to their levels of life satisfaction. People with PD score systematically higher than control subjects in all class intervals but the two lowest. Observed differences indicate that the item does not work the same way in the two groups. For comparison, panel B represents an item without DIF (item 4). DIF, Differential Item Functioning; PD, Parkinson’s disease; SWLS, Satisfaction with Life Scale

There was no DIF by time, age or gender, but item 5 exhibited uniform DIF by group. That is, except for in the lowest class intervals (those reporting lowest LS), PwPD were more likely to score higher than control subject on item 5 regardless of their levels of LS (Fig 4). Item 5 was then adjusted for the observed DIF by splitting it into two new subgroup specific items, one for PwPD and one for controls. The clinical significance of the observed DIF was then studied by assessing if the estimated person locations (logit measures) were affected by DIF. Person locations obtained after adjustment for DIF were compared to those estimated from the original non-DIF-adjusted scale. Before doing so, items without DIF in the original scale were anchored by their item locations from the DIF-adjusted scale to assure that the two sets of person estimates shared the same unit of measurement. The two resulting sets of person locations were very similar, with mean (95% CI) values of 1.07 (0.92–1.21) and 1.10 (0.95–1.25) logits for the unadjusted and DIF-adjusted scales, respectively. The intraclass correlation between the two was 0.998. In addition and also due to the observed signs of misfit of item 5, we explored the effects of omitting item 5. This compromised overall model fit (overall item-trait chi-square interaction, 49.04, P = 0.04) and yielded similar person locations (mean (95% CI), 1.34 (1.16–1.51) logits). The intraclass correlation between scales with and without item 5 was 0.950. Based on these observations item 5 was retained.

Separate Rasch analyses among PwPD and controls yielded very similar results to those obtained in the main analysis (Tables 3 and 4), including issues with the seven response categories. There was no DIF by time, age, gender or PD severity (according to UPDRS III groups) among patients with PD, whereas data suggested uniform DIF by gender for items 2 and 5 among control subjects. Adjustment for gender DIF by splitting item 5 removed the gender DIF associated with item 2, suggesting that this DIF was artificial. Similarly to the DIF by group in the main analyses, the gender DIF of item 5 did not have an appreciable influence on person estimates.

**Fig 5:**
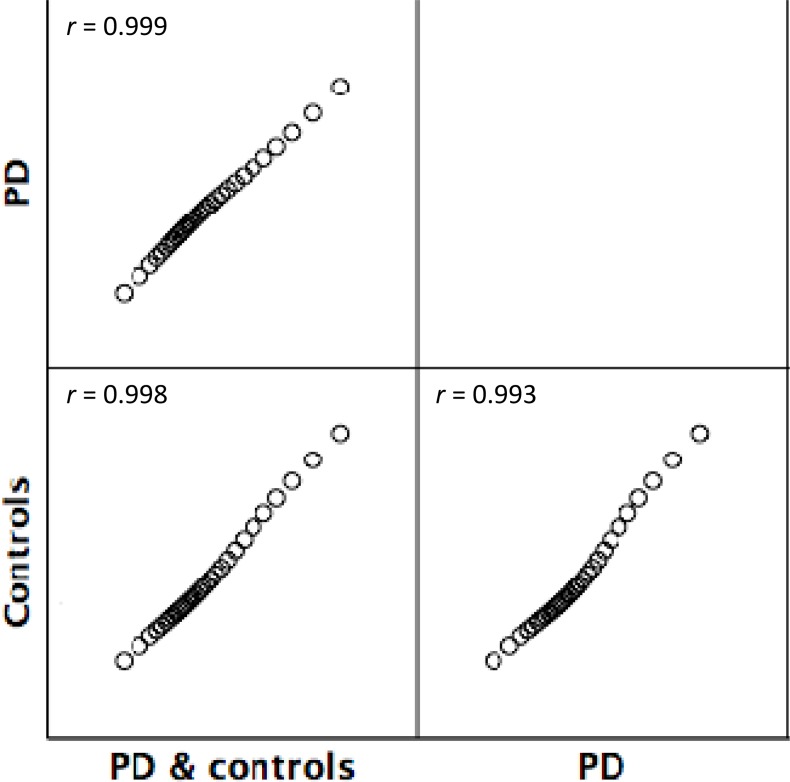
Scatterplot of the independently estimated linear logit locations. Matrix scatterplot of the independently estimated linear logit locations associated with each possible raw total SWLS score from the pooled sample (people with PD & controls), people with PD and control subjects (see Table 5). Inserted in each panel are the respective Pearson product-moment correlations (*r*). Nonparametric Spearman correlations were 1.0 in all three instances. Intraclass correlation across the three sets of estimates is 0.990. PD, Parkinson’s disease; SWLS, Satisfaction with Life Scale.

To further explore the measurement invariance of the SWLS as estimated for the pooled sample as well as separately for PwPD and control subjects, the linear logit locations associated with each possible raw total score were examined and are displayed in Table 5 together with the estimated standard error for each location. Fig 5 illustrates the relationships between these estimated logit locations from the three analyses.

**Table 5:** Interval level logit locations (measures) and standard errors associated with each possible ordinal level raw total SWLS score for the pooled sample (PwPD and controls) and for PwPD and controls separately^a^.

	PwPD & Controls		PwPD		Controls	
Raw total score	Location	SE	Location	SE	Location	SE
5	-3.228	1.071	-3.226	1.061	-3.125	0.999
6	-2.591	0.731	-2.577	0.747	-2.556	0.689
7	-2.180	0.567	-2.151	0.585	-2.193	0.534
8	-1.913	0.487	-1.871	0.501	-1.962	0.458
9	-1.713	0.437	-1.658	0.450	-1.788	0.411
10	-1.549	0.402	-1.483	0.415	-1.644	0.381
11	-1.406	0.379	-1.331	0.389	-1.519	0.361
12	-1.278	0.363	-1.194	0.372	-1.407	0.345
13	-1.160	0.351	-1.068	0.359	-1.302	0.335
14	-1.050	0.342	-0.950	0.35	-1.202	0.329
15	-0.943	0.337	-0.837	0.344	-1.104	0.326
16	-0.839	0.334	-0.729	0.339	-1.007	0.326
17	-0.735	0.334	-0.621	0.338	-0.908	0.328
18	-0.631	0.337	-0.514	0.339	-0.808	0.333
19	-0.523	0.341	-0.405	0.342	-0.702	0.341
20	-0.411	0.349	-0.293	0.347	-0.589	0.351
21	-0.293	0.359	-0.176	0.355	-0.467	0.365
22	-0.164	0.373	-0.051	0.366	-0.332	0.383
23	-0.022	0.391	0.083	0.380	-0.178	0.406
24	0.137	0.413	0.231	0.397	0.000	0.436
25	0.320	0.440	0.396	0.417	0.212	0.472
26	0.533	0.471	0.581	0.441	0.468	0.515
27	0.781	0.507	0.792	0.468	0.774	0.561
28	1.071	0.547	1.031	0.499	1.136	0.610
29	1.404	0.588	1.302	0.535	1.554	0.656
30	1.784	0.630	1.613	0.576	2.030	0.700
31	2.214	0.679	1.975	0.628	2.556	0.742
32	2.710	0.742	2.409	0.696	3.133	0.794
33	3.305	0.837	2.955	0.802	3.789	0.876
34	4.091	1.021	3.704	0.993	4.613	1.049
35	5.130	1.379	4.732	1.340	5.649	1.412

^a^ Based on Rasch analysis of data from people without any missing item responses, separately for the pooled sample (PwPD & controls; n = 536), PwPD (n = 253) and controls (n = 283).

SWLS, Satisfaction With Life Scale; PwPD, People with Parkinson’s disease; SE, standard error.

## Discussion

In this paper we have replicated previous CTT based psychometric results from using the SWLS in PD. We also expanded the analyses to account for the ordinal nature of item data in the CTT based analyses and to include tests according to Rasch measurement theory and comparability of the scale when used among PwPD and age-matched controls. There was a relatively high level of missing responses to the SWLS in the PwPD group resulting in 13% of noncomputable total SWLS scores. However, this is a lower rate than the 44% non-response rate reported by Rosengren et al. [8] and appears to be explained by our inclusion of people with cognitive impairments, as indicated by relatively lower scores the MMSE in the group with missing item responses (data not reported). However, our CTT based observations are in accord with previous reports and support the legitimacy of creating simple sum scores from the five SWLS items representing a common latent variable that is measured with acceptable levels of reliability and precision, both among PwPD and control subjects. Rasch analyses provided similar implications but yielded additional and new insights into the performance of the SWLS. Particularly, we were able to reveal problems related to the distinction of aspects of LS into seven rating scale categories. However, our study shows that the SWLS is a valid instrument for measuring LS in PD and in comparing PwPD with healthy individuals. Rasch analyses also illustrated that people reporting “extremely” high and low LS (according to Diener’s interpretation guide [29]) are measured with compromised precision. However, this is not considered a major problem because precision is arguably of less concern at the highest and lowest levels of LS, although it affects the ability to detect changes and differences within these levels. Furthermore, reliability was acceptable and the scale was still able to differentiate between 4–6 distinct strata of people.

The obvious way to improve targeting and precision would be to increase the number of items and/or response categories to enhance representation of the latent LS continuum. However, the brevity of the scale may be considered as one of its advantages from a practical point of view, and we found clear evidence that the number of response categories would need to be reduced, not increased. That is, respondents appear to have problems distinguishing between response categories expressing lower levels of LS. Collapsing the response categories from a seven- into a five-grade response scale seems reasonable in order to reduce this problem without compromising its reliability and (therefore) precision. However, this is to be considered an experimental procedure and we do not recommend relying on collapsed response categories since it is not known how people actually would have responded according to the collapsed categories [17, 28]. Instead, this needs to be examined empirically. Furthermore, it has been shown that collapsing categories that actually do work (albeit marginally, such as found here) can undermine the Rasch model [28], as illustrated here by compromised model fit following reduction of the response categories across all five items.

Fit of the SWLS to the Rasch model was generally acceptable. Some of the statistical indices of model fit such as the total item-trait interaction chi-square based P-value and fit residuals in some cases exhibited values outside generally recommended ranges. However, it should be noted that there is no single aspect of fit that is either necessary or sufficient for the evaluation of fit, but all data need to be considered relatively, interactively and in perspective of context [17, 19, 20]. Indeed, as evident from the graphical representations of item model fit, empirical item responses exhibited close accordance with model expectations. The possible exception was item 5, which exhibited a pattern suggestive of multidimensionality. This is in accordance with previous reports suggesting that this item (together with item 4) may represent a somewhat different dimension than the other SWLS items due to referring to the past, as opposed to the present [29]. Considering the item wording, this is more evident for item 5 than for item 4, which is in accordance with our observations in that it was item 5 that exhibited signs of misfit. However, the misfit of item 5 was relatively modest, its deletion did not improve the scale, EFA supported unidimensionality, and assessment of the two suggested subdimensions of the SWLS did not reveal evidence of multidimensionality since person location estimates did not differ in more instances than would be expected by chance.

We also found that item 5 was associated with DIF by group in the main analysis and by gender among control subjects. While this is an additional indication that this item is not entirely coherent with the other SWLS items, the observed DIF did not appear to cause any obvious bias to the SWLS as a measure of LS. Therefore, taken together and given the theoretical underpinnings of its construction, the SWLS appears to exhibit reasonable enough fit to the Rasch model to provide measurement of LS among PwPD and age-matched controls that is useful for most circumstances. However, our data also show that in addition to the seven-grade SWLS response scale, reconsideration of content and/or wording of item 5 may be worthwile in future attempts to improve the scale.

The SWLS item hierarchy, i.e., the ordering of items from lower to higher LS according to their logit locations was consistent across the samples with regard to items representing the lowest and highest levels of LS (items 4 and 1, respectively). Furthermore, taking the uncertainty (i.e., item location standard errors) associated with the estimated locations into account, the hierarchies of the other items did not exhibit any clear differences between the samples. The hierarchy also appears to make general theoretical and clinical sense in that considering one’s life as close to ideal (item 1) represents higher levels of LS than it does to agree that one has achieved the important things in life (item 4). This provides general support for the internal construct validity of the SWLS [17].

## Conclusion

We replicated previous psychometric CTT based results and expanded the analyses by taking account of the ordinal nature of item responses and using Rasch measurement theory. Rasch analyses illuminated new aspects and more detailed information regarding the performance and validity of the SWLS, and identified areas for future implications in order to improve the scale. In particular, future studies should try to confirm whether the scale would benefit from a reduction from seven to five response categories. However, our findings support the SWLS as a reliable and valid instrument for measuring LS in PwPD, and that the scale is able to distinguish between levels of LS in and between PwPD and healthy controls. These observations are of considerable significance as life satisfaction and related constructs are central to a person-centred approach to chronic disease management.
